# Effects of Cigarette Smoking on Retinal and Choroidal Thickness: A Systematic Review and Meta-Analysis

**DOI:** 10.1155/2019/8079127

**Published:** 2019-09-29

**Authors:** Tian-Ke Yang, Xiao-Gang Huang, Jing-Yan Yao

**Affiliations:** Department of Ophthalmology, First Hospital Affiliated to Soochow University, Suzhou, Jiangsu Province, China

## Abstract

**Background:**

Cigarette smoking has been regarded as a risk factor for the incidence of a wide variety of chronic illness; however, its effect on thickness of the retina or choroid is still unknown.

**Methods:**

A consummate literature search was conducted in PubMed and Embase up to January, 2018. The quantitative synthesis was conducted by Stata 12.0.

**Results:**

A total of 13 observational studies were included in this meta-analysis. In this meta-analysis of all available observational studies, no significant effect of tobacco smoking on retinal or choroidal thickness change was detected. However, advanced analyses showed that smoking would influence the thickness of RNFL (average: SMD, −0.332; 95% CI, −0.637 to −0.027; inferior: SMD, −0.632; 95% CI, −1.092 to −0.172; and superior: SMD, −0.720; 95% CI, −0.977 to −0.463) and GCL (superior: SMD, −0.549; 95% CI, −0.884 to −0.215; inferior: SMD, −0.602; 95% CI, −0.938 to −0.265). Meanwhile, subgroup analyses demonstrated that the results based on studies in some regions (America and Africa) and cross-sectional studies showed a reduced choroidal thickness in smokers. No publication bias was detected in this study.

**Conclusion:**

In conclusion, no significant effect of tobacco smoking on retinal or choroidal thickness change was detected. However, smoking would influence the thickness of RNFL and GCL. Future research on this field would help in the prevention and treatment of smoking-associated disorders.

## 1. Introduction

Cigarette smoking has been regarded as a risk factor for the incidence of a wide variety of chronic illness, including kinds of carcinomas [1], respiratory disorders [2], cardiovascular disease [3], and other systemic diseases. In general, it was considered that mortality among current smokers would be up to 2 to 3 times as that among persons who never smoked [4]. As a highly modifiable environmental factor, smoking was reported to be associated with kinds of ocular disorders, including age-related macular degeneration (AMD), glaucoma, ischemic optic neuropathy, and retinal vein occlusion [5–8]. With mounting evidence to suggest that tobacco smoking is a causative factor in ocular diseases, the question of the contribution of smoking to disease pathology also requires advanced examination. In an experiment based on both *in vitro* and *in vivo* studies, it was found that impaired retinal pigment epithelium- (RPE-) derived MCP-1-mediated scavenging macrophages recruitment and phagocytosis might lead to incomplete clearance of proinflammatory debris and infiltration of proangiogenic macrophages which might promote the progression to choroidal neovascularization in smoker patients with dry AMD [9]. In another study, it was found that nicotine would increase the VEGF/PEDF ratio in the RPE through nAchR *in vitro* and *in vivo* [10]. In other studies, it was reported that smoking impacts vascular endothelium by increasing oxidative stress, decreasing antioxidant vitamin C level, and inducing abnormal nitric oxide activity [11].

Considering that the anatomy and physiologic functions of the choroid and retina were associated with the smoking-related disorders, such as AMD and glaucoma, the changes of choroidal and retinal thickness in smokers might explain the pathogenesis. Spectral-domain optical coherence tomography (SD-OCT) has been used to acquire high-resolution scans of the retina and choroid, and thus, it is a noninvasive *in vivo* measurement of the retinal and choroidal thickness. In the Hisayama study, the factors associated with foveal thickness (FT) and macular thickness (MT) were examined. Through multiple linear regression analysis, it was found that current smoking was significantly associated with FT and MT [12]. However, the effect of smoking on the thickness of the choroid and retina was quite discordant in previous studies. Meta-analysis is an effective statistical tool to combine consubstantiate but independent studies and thus gain a more powerful conclusion. The aim of this study is evaluate the effect of smoking on choroidal and retinal thickness through a meta-analysis of available evidence.

## 2. Materials and Methods

### 2.1. Sources, Search Criteria, and Data Extraction

This meta-analysis was conducted following recommendations made by both Preferred Reporting Items for Systematic Reviews and Meta-Analysis (PRISRMA) [13] and the Meta-analysis of Observational Studies in Epidemiology group (MOOSE) [14]. Observational studies, including cross-sectional and case-control studies, about the effects of smoking on retinal or choroidal thickness change were included in this meta-analysis. Two reviewers conducted a systematic review of the literature in PubMed and Embase up to January 2018. The following keywords “smoking,” “cigarette,” “tobacco,” and “nicotine” in combination with “retinal thickness,” “choroidal thickness,” “retinal nerve fiber layers,” and “ganglion cell layer” were used for the detection of potentially relevant studies. In addition, the reference lists of relevant reviews were checked for additional publications. No language or other restrictions were imposed in the literature search.

The relevant studies would be considered inclusion if they met the following inclusion criteria: (a) an observational study design was obtained (cross-sectional, case-control, or cohort study); (b) the effect of smoking on retinal or choroidal thickness was detected; (c) the detailed value of thickness of retina/choroid was provided, or original data that could calculate them were reported. The study inclusion was conducted by two reviewers independently (TK Yang and JY Yao). Any disagreements would be resolved by discussion with the third reviewer (XG Huang).

Data extraction was conducted by two reviewers independently (T. K. Yang and J. Y. Yao). The extracted data were recorded in a standardized data-collection form. The following information in each included study was extracted and recorded: name of the first author, publication date, study location, study design, amount, gender, age of all the participants, thickness of retina/choroid detection methods, smoking definition, and confounding factors adjusting status. And, mean value with standard deviation (SD) was extracted or calculated. Any disagreements would be resolved by discussion with the third reviewer (X. G. Huang).

### 2.2. Quality Assessment

Considering the observational study nature of the included studies, we developed a modified scoring scale for this study based on the Newcastle–Ottawa scale (NOS), which is a 9-star scale designed for case-control and cohort study [15]. The scale system was as following, and one point was allocated for one item: (1) a prospective study design was adopted; (2) a case-control study was adopted; (3) appropriate inclusion and exclusion criteria for all the participants were provided; (4) general characteristics, such as age and sex, in all the participant population were matched; (5) other potential confounding factors, such as refractive error and axial length, were matched; (6) the smoking group definition was reasonable and (7) described the details of the control group; (8) OCT was obtained for the thickness of retina/choroid detection; (9) the detailed detection method and progression were described. A higher score indicated a high quality. In this study, high-quality studies were defined as quality score over 6 points. The quality assessment was conducted by two reviewers independently (T. K. Yang and J. Y. Yao). Any disagreements would be resolved by discussion with the third reviewer (X. G. Huang).

### 2.3. Statistical Analysis

The effect of tobacco smoking on the thickness of the retina or choroid was detected by pooling the data from the included studies. Pooled standard mean difference (SMD) with 95% CIs would be used for data presentation in forest figures. Presence of heterogeneity in effects was assessed using both *χ*^2^ test and *I*^2^ test. The *P* < 0.10 level or *I*^2^ > 50% would be considered as significance of heterogeneity. As only observational studies were included in this study, a random-effect model was obtained in this current meta-analysis. To explore the possible source of heterogeneity, advanced subgroup analysis stratified by study designs, various structures, and detection equipment would be conducted to assess the potential influence on overall outcome and significance of heterogeneity.

To detect the robustness of the conclusion in this study, the sensitivity analysis was conducted by two independent methods. First, one study would be dropped from all the included studies in each turn, thus investigating the influence of a single study on the overall risk. Secondly, we also removed the studies with relatively lower quality (quality score less than 5 points), and the outcome was obtained to estimate the reliability of the conclusion in this study. Considering that funnel plots have several limitations in the detection of publication bias, both Begg's and Egger's tests were conducted in the testing of publication bias. The STATA software (version 12.0; StataCorp LP) was used in the statistical analysis. A *P* value less than 0.05 was considered statistically significant, except where otherwise specified.

## 3. Results

### 3.1. Literature Search

**Figure 1:**
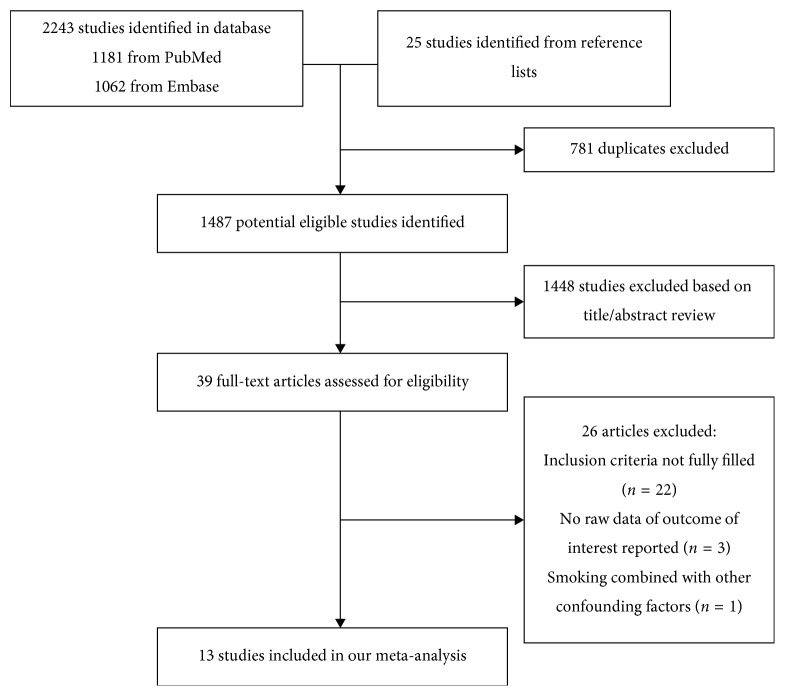
The diagram for identification of relevant studies.

The flow diagram identifying the relevant studies in this study is presented in Figure 1. A total of 2243 articles were identified from the three databases search (1181 form PubMed and 1062 from Embase). Furthermore, 25 records were selected from the reference lists of the relevant articles. After excluding 781 duplicates, a total of 1487 articles were identified for potential inclusion. Through title and abstract review, 1448 studies were removed and 39 full-text articles were assessed for eligibility. In final, a total of 13 studies were included in this meta-analysis after 26 records were excluded (22 studies did not fill the inclusion criteria, 3 studies did not report the outcome of interest, and one study confused smoking was with other factors).

### 3.2. Study Characteristics

In this study, a total of 13 observational studies (7 case-control, 5 cross-sectional, and 1 prospective consecutive case series study) were included [16–28]. In the included studies, we identified a total of 614 smokers and 625 controls. The publication dates of all the studies ranged from 2013 to 2017. Among all the included studies, 10 studies were in Europe, 1 study was in America, and the last 2 studies in Africa. The average score of methodological scale was 5.69 ± 0.85 (ranging from 4 to 7 points). Most included studies demonstrated a relatively high methodological quality (over 5 points). The detailed characteristics of all the studies included in the final analysis are presented in Table 1.

### 3.3. Effects of Smoking on Thickness of Retina and Choroid

**Figure 2:**
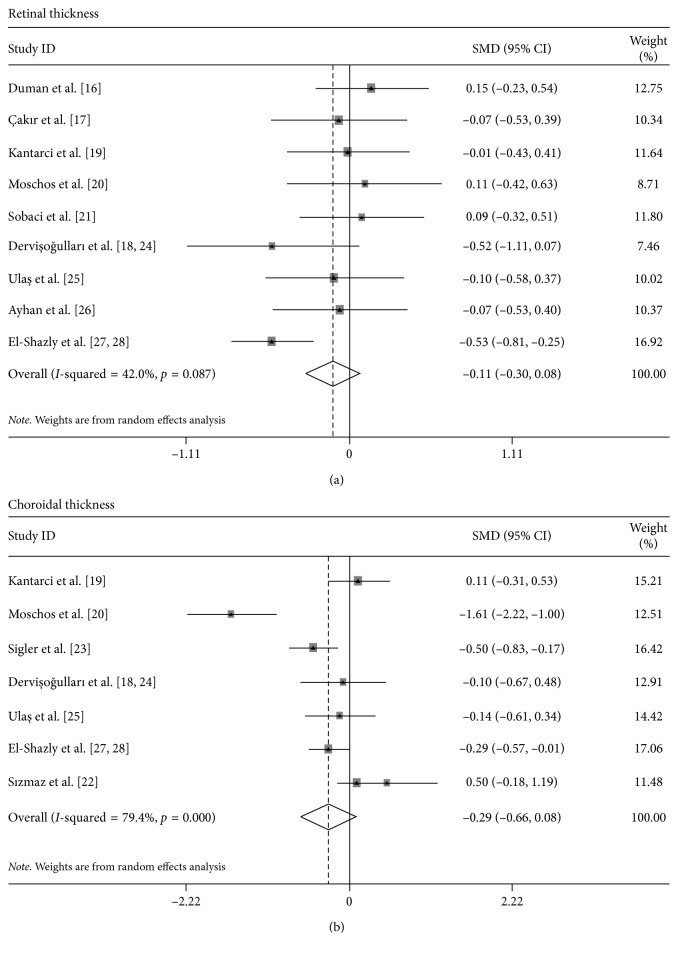
Forest plot for the effect of smoking on the thickness of (a) retina and (b) choroid.

Through pooling all the included studies together, it was found that there was neither significant difference in retinal thickness (SMD, −0.11; 95% CI, −0.30 to 0.08, Figure 2(a)) nor choroidal thickness (SMD, −0.29; 95% CI, −0.66 to 0.08, Figure 2(b)).

To gain more available information from the included studies, we performed a series of subgroup analyses. When the retina was considered, three different layers, including central retina, retinal nerve fiber layer (RNFL), and ganglion cell layer (GCL), were reported most frequently in previous studies. In the central retinal thickness detection, the subgroup analyses by regions, study designs, and detection equipment showed that only the studies in Africa showed a decreased central retinal thickness in smokers (SMD, −0.528; 95% CI, −0.810 to −0.246). However, it was found that the average (SMD, −0.332; 95% CI, −0.637 to −0.027), inferior (SMD, −0.632; 95% CI, −1.092 to −0.172), and superior (SMD, −0.720; 95% CI, −0.977 to −0.463) in RNFL thickness demonstrated a significant reduction in smokers. When the GCL thickness was considered, only superior (SMD, −0.549; 95% CI, −0.884 to −0.215) and inferior (SMD, −0.602; 95% CI, −0.938 to −0.265) demonstrated a statistical difference.

We also conduct several subgroup analyses of the effect of tobacco smoking on choroidal thickness. It was found that it is in America (SMD, −0.499; 95% CI, −0.829 to −0.170) and Africa (SMD, −0.291; 95% CI, −0.570 to −0.012) but not Europe (SMD, −0.195; 95% CI, −0.431 to 0.041) demonstrated a significant decrease in choroidal thickness. When the study design was considered, the significant outcome was detected in the cross-sectional group (SMD, −0.228; 95% CI, −0.450 to −0.006) but not the case-control group (SMD, −0.329; 95% CI, −1.517 to 0.859). We also conduct a subgroup analyses by detection equipment; however, no significant outcome was detected in any groups (Heidelberg, Cirrus, and other groups). The detailed subgroup analysis effect of smoking on the thickness of the retina and choroid is presented in Table 2.

### 3.4. Heterogeneity and Sensitivity Analysis

Statistically significant heterogeneity was detected in both retinal thickness (*I*^2^, 42.0%; *P* < 0.087) and choroidal thickness (*I*^2^, 79.4%; *P* < 0.001). Advanced subgroup analysis did not provide good results in the heterogeneity detection. Considering that a random-effect model was adopted in assessing the pooled effect when the heterogeneity is significant and a relatively conservative conclusion was obtained, the significant heterogeneity might not influence reliability of the conclusion in this study.

To conduct the sensitivity analysis, both one-way sensitivity analysis and sensitivity analysis based on methodological scales were obtained. In the retinal thickness section, the results from one-way analysis and sensitivity analysis based on methodological scales demonstrated little change in the quantitative summary measure of SMD with 95% CI, and there was no any single study influencing conclusion of tobacco smoking on retinal thickness.

However, the one-way sensitivity analysis showed that when the work by Sızmaz et al. [22] was removed from the meta-analyses, the outcome turns to be significant (SMD, −0.390; 95% CI, −0.762 to −0.017). After dropping the studies with relatively lower methodological scales, no significant positive effect was also detected (SMD, −0.249; 95% CI, −0.701 to 0.203).

### 3.5. Publication Bias

**Figure 3:**
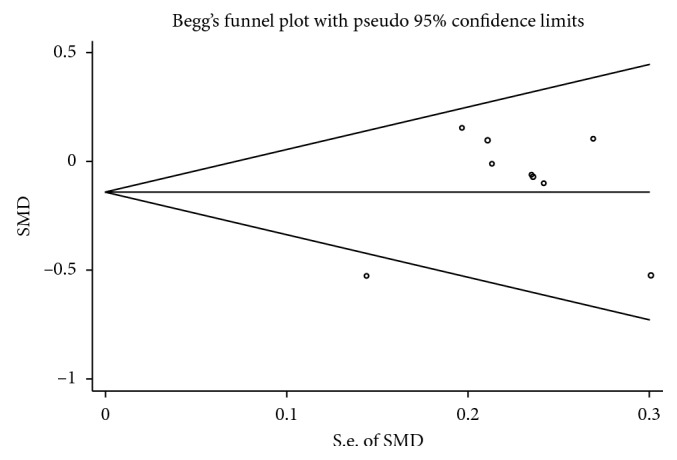
Funnel plot for assessment of publication bias.

In this study, there was litter evidence of a publication bias obtained by neither the Begg funnel plot (*P*=0.251, Figure 3) nor the Egger's test (*P*=0.222).

## 4. Discussion

In this meta-analysis of all available observational studies, no significant effect of tobacco smoking on retinal or choroidal thickness change was detected. However, advanced analyses showed that smoking would influence the thickness of RNFL and GCL. In addition, subgroup analyses demonstrated that the results based on studies in some regions (America and Africa) and cross-sectional studies showed a reduced choroidal thickness in smokers. No publication bias was detected in this study; however, sensitivity analysis on the choroidal thickness section showed that more related studies were required in the future.

The harmful effect of tobacco on kinds of chronic diseases was generally accepted in previous literatures [29], and poor understanding was gained about the effect of tobacco smoking on the anatomical change in the retina or choroid. As a common modifiable lifestyle, the recognition of the effect of tobacco smoking on retinal or choroidal thickness change would provide advanced knowledge on pathogenesis of smoking-associated ocular diseases as well as improved medical suggestions on their prevention. Several previous studies focused on other points in related fields. Through optical coherence tomography angiography (OCTA) methods, Ayhan et al. and collaborator reported the effect of smoking on macular perfusion. The OCTA measurements were conducted at baseline, 5 minutes, 30 minutes, and 60 minutes after one standard cigarette smoking, and the results showed that smoking would cause a significant decrease in the blood flow index of the choriocapillary area by the acute effects of nicotine and other chemical substances in cigarettes [26]. In another study, the microstructural and functional changes in the macula of heavy habitual smokers were also reported. It was found that decreased macular autofluorescent pigment density (MAPD) and altered response to photostress would be indicative of early nicotine toxicity in microstructurally sound macula of adult chronic smokers [21].

However, it was hard to conduct a randomized controlled trial to study the effect of smoking intervention on long-term retinal or choroidal thickness change, and thus, previous evidences focused on the observational findings in this field. Meta-analysis is a useful statistical tool, which could pool consubstantiate but independent studies together, would provide larger power, and obtain a systematic evaluation. Considering that the data regarding smoking and retinal/choroidal thickness change were less convincing by now, it was suitable to conduct a meta-analysis on observational data on this issue. In this meta-analysis, it was found that no significant effect of tobacco smoking on retinal or choroidal thickness change was detected. However, a significant decrease of RNFL and GCL was detected. It has been long recognized that tobacco smoking was associated with nervous tissue damage and would influence the recovery of normal repair of nerve injury [30]. Through evaluating the visual field changes in 24 healthy, chronic, heavy cigarette smokers and 16 age- and sex-matched healthy nonsmokers, it was found that chronic, heavy cigarette smokers would decrease retinal sensitivity and this might affect the retina or optic nerve functions cumulatively [31]. Considering the previous researches, it was quite natural to conjecture that smoking would influence that optic nerve maintenance. Several potential associated mechanisms existed could explain this phenomenon. Firstly, chronic nicotine toxicity by the direct neurotoxic effect on the optic nerve might explain the impairment and thus could also explain that more range of RNFL than GCL were impaired in this study. Secondly, as mentioned above, nicotine or other chemical materials would decrease the volume of blood flow in peripheral circulation and thus lead to an aggravated nerve function. Third, it was reported that smoking history was associated with higher intraocular pressure [32], and it also might be associated with lower RNFL and GCL. However, considering the small amount included studies in this study, it was hard to conduct more detailed analyses. Besides, far more advanced study, especially well-designed prospective studies with detailed functional examination, would provide more knowledge.

Even no significant influence in the choroidal thickness in the smokers was detected in this study, the subgroup analysis by region and study designs provided different outcomes. Moreover, the sensitivity analyses showed that the conclusion in this point was influenced by one included study, and thus, more relevant studies were required in the future. However, the result in this study also highlighted the potential importance of the effect of smoking on the choroidal thickness. Choroid is present between the retina and the sclera, and it contains rich blood vessels and pigment cells. Smoking, which is recognized as a factor influencing the vascular endothelial cell function [33], could influence the choroid as well. Besides, considering that retinal vascular reactivity in response to normoxic hypercapnia is significantly reduced in young, healthy smokers compared with nonsmokers [34], the reactivity of the choroidal vessel could also be influenced. However, the choroidal thickness was influenced by kinds of factors, such as systemic blood pressure, ocular parameter, and aging change, and the confident conclusion was required in the following studies.

There were several strengths in this current meta-analysis. First, the comprehensive literature search strategy would help us to obtain all available studies. The inclusion of all potential observational studies would help in the overall recognition and detailed analysis on the effect of smoking on retinal/choroidal thickness change. Second, detailed subgroup analyses would provide us better knowledge and give the guide for following study design. There were several limitations of this meta-analysis should be acknowledged. First, the differences in the designs (case-control, cross-sectional, and case series) would influence the widely accepted conclusion studies. The disconfirmation in the effect of smoking on retinal and choroidal thickness demonstrated that the conclusion should be validated in more well-designed studies. Secondly, the results of this study were based on observational studies, and it must be pointed out that the evidence of this study was insufficient to identify the effect of smoking on retinal/choroidal thickness. Third, the sensitivity analyses on the choroidal thickness section demonstrated inconsistent conclusion, and it might affect the reliability of the conclusion in this study. However, considering the relative small amount of the included studies in this analyses, advanced well-designed studies would help in the better understanding on this issue.

In conclusion, no significant effect of tobacco smoking on retinal or choroidal thickness change was detected. However, smoking would influence the thickness of RNFL and GCL. Furthermore, subgroup analyses demonstrated that the results based on studies in some regions (America and Africa) and cross-sectional studies showed a reduced choroidal thickness in smokers. For smoking-related chorioretinal diseases, the conclusions of this research provides ideas for follow-up studies. In addition, future research on this interesting and meaningful point would help in the prevention and treatment of smoking-associated disorders.

## Figures and Tables

**Table 1:** Characteristics of all the included studies regarding the effect of smoking on the thickness of retina and choroid.

Author, year	Study design	Country	No. of case/control	Age (years)	Gender (female/male)	Retina and choroid detection	Smoking definition	Adjustments/matched	Scale^#^
Duman et al., 2017	Cross-sectional	Turkey	54/54	40.13 ± 12.36/40.57 ± 12.13	52/56	Heidelberg Spectralis	10–80 pack-years	Age and gender	5
Çakır et al., 2016	Case-control	Turkey	36/36	26.2 ± 6.0/25.7 ± 4.1	30/42	Cirrus EDI-OCT	20 cigarettes/day for 5 years	Age and gender	6
Dervişoğulları et al., 2015	Case-control	Turkey	44/44	39.85 ± 8.41/38.66 ± 10.47	20/68	Cirrus HD-OCT	≥1 pack of cigarettes/day for >10 years	Age and gender	7
Kantarci et al., 2016	Case-control	Turkey	46/42	51.8 ± 8.5/48.3 ± 9.9	15/73	RS-3000 OCT Retina Scan	10–120 pack-years	Age, sex, spherical equivalent, and AL	5
Moschos et al., 2016	Case-control	Greece	31/25	57.8 ± 4.5/68.0 ± 4.1	25/31	Heidelberg Spectralis	>25-year cigarette smoking	Age and gender	5
Sobaci et al., 2013	Case-control	Turkey	45/45	32.9 ± 3.9/33.1 ± 4.1	33/57	Heidelberg retinal angiogram	>1 box/day for >5 years	Age and gender	6
Sızmaz et al., 2013	Case-control	Turkey	17/17	34.3 ± 8.0/31.3 ± 5.9	16/18	Optovue Inc	3–45 cigarettes/day	Age and gender	6
Sigler et al., 2014	Prospective consecutive case series	USA	67/80	78 ± 7.2	NA	Heidelberg Spectralis	Current and former smoking	NA	4
Dervişoğulları et al., 2015	Prospective cross-sectional	Turkey	24/22	38.62 ± 8.86/34.40 ± 8.76	10/36	Cirrus HD OCT	Smoking for over 10 years	Age and gender	5
Ulaş et al., 2014	Cross-sectional	Turkey	30/40	28.73 ± 3.75/29.75 ± 2.90	35/35	Spectralis SD OCT	20–40 cigarettes/day for 1 year	Age and gender	6
Ayhan et al., 2017	Case-control	Turkey	40/40	41.2 ± 9.1/42.6 ± 8.4	24/56	XR Avanti AngioVue OCTA	Chronic smoking	Age and gender	6
El-Shazly et al., 2017	Cross-sectional	Egypt	80/80	26.28 ± 3.83/25.53 ± 3.90	11/149	Retina Scan RS-3000 Advance	≥10 cigarettes for 10 years	Age and gender	6
El-Shazly et al., 2017	Cross-sectional	Egypt	100/100	27.41 ± 4.71/26.41 ± 4.33	0/200	Retina Scan RS-3000 advance	Smoking for over 10 years	Age and gender	7

^#^A modified scoring scale for this study based on Newcastle–Ottawa scale (NOS).

**Table 2:** Subgroup analysis of effect of smoking on the thickness of retina and choroid.

Outcome of interest	Subgroups	No. of studies	Summary effect		Heterogeneity	
			SMD (95% CI)	*P* value	*I* ^2^, %	*P* value
Retina	Central retina					
	Region					
	Europe	8	−0.019 (−0.193 to 0.156)	0.833	0	0.636
	Africa	**1**	−**0.528 (**−**0.810 to** −**0.246)**	**<0.001**	—	—
	Study design					
	Case-control	5	−0.246 (−0.606 to 0.114)	0.180	66.8	0.029
	Cross-sectional	4	0.016 (−0.214 to 0.247)	0.889	0	0.919
	Detection					
	Heidelberg	3	0.074 (−0.146 to 0.294)	0.511	0	0.869
	Cirrus	2	−0.242 (−0.606 to 0.121)	0.192	29.6	0.233
	Others	4	−0.334 (−0.782 to 0.114)	0.249	47.6	0.064
	Retinal nerve fiber layer					
	Average	3	−**0.332 (**−**0.637 to** −**0.027)**	**0.033**	58.8	0.063
	Inferior	2	−**0.632 (**−**1.092 to** −**0.172)**	**0.007**	66.9	0.082
	Superior	2	−**0.720 (**−**0.977 to** −**0.463)**	**<0.001**	0	0.886
	Nasal	2	−0.406 (−1.161 to 0.349)	0.292	87.5	0.005
	Temporal	2	−1.621 (−4.402 to 1.161)	0.253	98.5	<0.001
	Ganglion cell layer					
	Average	2	−0.229 (−0.470 to 0.012)	0.062	15.4	0.307
	Superotemporal	1	−0.341 (−0.762 to 0.080)	0.112	—	—
	Superior	2	−**0.549 (**−**0.884 to** −**0.215)**	**0.001**	51.8	0.15
	Superonasal	1	−0.222 (−0.641 to 0.198)	0.300	—	—
	Inferotemporal	1	−0.104 (−0.522 to 0.314)	0.625	−	−
	Inferior	2	−**0.602 (**−**0.938 to** −**0.265)**	**< 0.001**	61	0.109
	Inferonasal	1	−0.228 (−0.647 to 0.191)	0.286	—	—
Choroid	Region					
	America	**1**	−**0.499 (**−**0.829 to** −**0.170)**	**0.003**	—	—
	Europe	5	−0.195 (−0.431 to 0.041)	0.105	85.2	<0.001
	Africa	**1**	−**0.291 (**−**0.570 to** −**0.012)**	**0.041**	—	—
	Study design					
	Case-control	3	−0.329 (−1.517 to 0.859)	0.587	92.5	<0.001
	Cross-sectional	3	−**0.228 (**−**0.450 to** −**0.006)**	**0.044**	0	0.762
	Detection					
	Heidelberg	2	−0.716 (−1.435 to 0.003)	0.051	86.3	0.001
	Cirrus	1	−0.096 (−0.675 to 0.483)	0.746	—	—
	Others	4	0.027 (−0.399 to 0.454)	0.9	65.5	0.055

SMD: standard mean difference; CI: confidence interval. The outcome marked as bold demonstrated significant effect.
